# Very late stent thrombosis with bare-metal stent: identifying severe stent malapposition and underexpansion by intravascular ultrasound

**DOI:** 10.1590/S1679-45082013000300017

**Published:** 2013

**Authors:** Adriano Caixeta, Vanessa Cristina Salomon Palma Braga, Gary S Mintz

**Affiliations:** 1 Hospital Israelita Albert Einstein, São Paulo, SP, Brazil; 2 Cardiovascular Research Foundation, New York, USA

**Keywords:** Stents, Myocardial infarction, Thrombosis/ultrasonography, Case reports

## Abstract

A 60-year-old man with a history of implantation of two bare-metal stents 2 years prior presented to the emergency department with new-onset chest pain. He has been regularly taking angiotensin-converting enzyme inhibitors, beta blockers and aspirin. Aspirin was suspended for 10 days prior to the current hospitalization in order to perform surgery to remove a kidney tumor. He underwent coronary angiography, which revealed a right coronary artery with a distal intraluminal defect within the stents, suggesting thrombus. Intravascular ultrasound demonstrated a severe malapposition and underexpansion throughout the entire length of the stents containing thrombus. In this case, the mechanisms of very late stent thrombosis after bare-metal stent most likely were a combination of mechanical factors (severe stent undersizing during the index procedure) and pharmacological factors (aspirin discontinuation).

## RESUMO

Paciente de 60 anos de idade, com histórico de implante de dois *stents* coronários não farmacológicos durante quadro de infarto do miocárdio com supra do segmento-ST. Dois anos após, foi admitido no pronto-socorro com quadro clínico de angina instável de início recente. O paciente fazia uso regular de inibidor da enzima de conversão da angiotensina, betabloqueador e aspirina. A aspirina foi suspensa por 10 dias antes da atual hospitalização para a retirada de um tumor de rim. O paciente foi submetido a angiografia coronária, que revelou artéria coronária direita com defeito de enchimento intraluminal sugestivo de trombo intra-*stent*. A ultrassonografia intravascular demonstrou má aposição e expansão dos *stents*, além da presença de trombo. Neste caso, o mecanismo de trombose muito tardia de *stent* coronário não farmacológico deveu-se possivelmente a uma combinação de fatores mecânicos (implante de *stents* subdimensionados durante o procedimento índice) e farmacológico (descontinuação da aspirina).

## INTRODUCTION

Primary percutaneous coronary intervention with stent implantation has emerged as the treatment of choice for most patients with evolving ST-segment elevation myocardial infarction (STEMI). Nevertheless, emerging data suggest that risk of stent thrombosis in STEMI is relatively increased in patients without STEMI^(1)^ and occurs with similar frequency in bare-metal stent (BMS) and drug-eluting stents (DES)^(2)^. Endothelization and healing at the site of stent implantation in patients with STEMI may be delayed substantially^(3)^. Furthermore, although there is no difference in the frequency of stent thrombosis with both stents types, the relatively high very late stent thrombosis rate recently documented with BMS^(2)^ is notable and refuses the prior conception^(4)^ that, at least in STEMI, very late stent thrombosis is a rare phenomenon. In addition, data and imaging documenting the mechanism of very late stent thrombosis in STEMI patients is rare. The aim of this case was to report the potential mechanisms of very late stent thrombosis after BMS by intravascular ultrasound (IVUS).

## CASE REPORT

**Figure 1:**
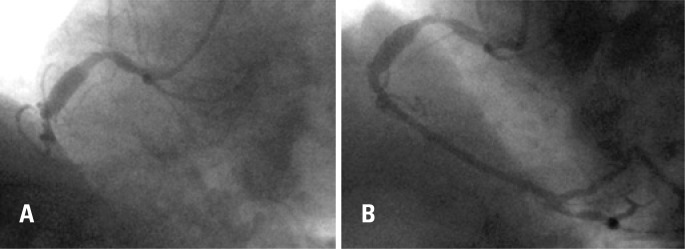
Coronary angiography showing the right coronary artery before (A) and after (B) primary coronary angioplasty and stenting 2 years prior to the current hospitalization

**Figure 2:**
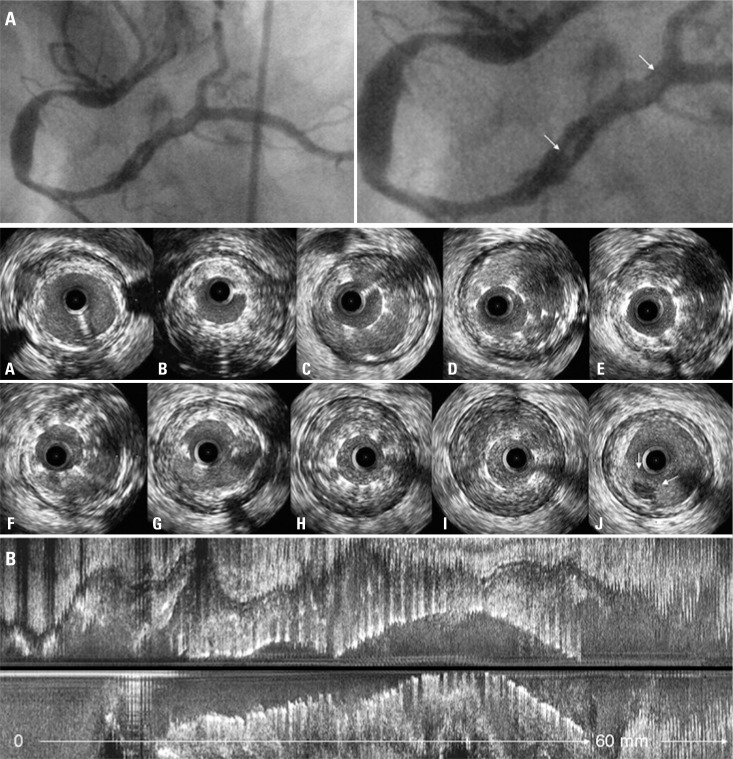
Diagnostic intravascular ultrasound was performed to assess the angiographic filling defect at the right coronary artery (A, white arrow in the angiography). The intravascular ultrasound images are shown from proximal (A) to distal (J) in figure B. There was severe malapposition and underexpansion throughout the entire length of the stents. Notice the space between the stent strut and the intima and the blood speckle/thrombus behind the stent struts in the axial (B through I) as well as the longitudinal view (at the bottom). At the site of maximum stent malapposition (I), the stent area (4.99mm^2^) was smaller than lumen area (15.22mm^2^) and external elastic membrane (26.64mm^2^). The entire distal 20+mm of the stent was thrombus filled with additional thrombus on the abluminal side of the stent partly filling the area of malapposition and causing the linear filling defect on the angiogram. The small intraluminal mass on IVUS (J, small arrows) represented the tail of the thrombus with the bulk being proximal to that slice

A 60-year-old man with a history of implantation of two BMS (2.25x28mm and 2.5x28mm) for an inferior STEMI (Figure 1) 2 years prior presented to the emergency department with new-onset chest pain. Electrocardiography showed new inferior wall changes and elevated serum creatine kinase-MB and troponin at 3.03ng/mL and 11.10ng/mL, respectively. He had been taken regularly angiotensin-converting enzyme inhibitors, beta blockers and aspirin at home, but the latter was suspended for 10 days prior to the current hospitalization in order to perform surgery to remove a kidney tumor. The patient underwent coronary angiography, which revealed a right coronary artery with diffuse ectasia containing two lesions (at proximal and mid right coronary artery) and a distal intraluminal defect within the stents (Figure 2A, white arrows), suggesting thrombus. The IVUS (iLab, Boston Scientific, USA) of the lesions demonstrated a distal large vessel with a severe malapposition and underexpansion throughout the entire length of the stents containing thrombus (Figure 2B).

## DISCUSSION

Although it is a relatively low-frequency event, stent thrombosis is associated with high risk of myocardial infarction and death^(5)^. The risk of stent thrombosis in the STEMI population is increased threefold compared with non-STEMI patients^(1)^, and occurs with similar frequency in BMS and DES^(2)^. The higher rate of stent thrombosis in patients with STEMI may be related to the underlying plaque composition responsible for the condition. Stent strut penetration of an underlying necrotic core may augment inflammation, platelet activation, and fibrin deposition and inhibit neointimal growth, resulting in uncovered stent struts^(3)^. Furthermore, the incidence of positive arterial remodeling and late acquired incomplete stent apposition is higher in patients with acute coronary syndromes, especially in those with DES. Finally, infarct-related artery vasoconstriction, reduced epicardial flow, and thrombus may lead to stent undersizing, as likely occurred in the current case at the time of STEMI treatment, when a 4.5mm vessel was treated with a 2.5mm stent. Over time, when flow was reestablished, thrombus dissolved, and the vessel increased in size, which developed malapposition and underexpansion of the stent. In this case, the mechanisms of very late stent thrombosis after BMS most likely were a combination of mechanical factors (severe stent undersizing during the index procedure with or without superimposed positive remodeling and infarct-related thrombus dissolution during the 2 year follow-up period) and pharmacological factors (aspirin discontinuation).

## CONCLUSION

This case reports the mechanisms of very late stent thrombosis 2 year after bare-metal stent implantation. Intravascular ultrasound plays an important role in identifying mechanical factors related to stent thrombosis as well as guiding its therapy accordingly.
